# Wealth, health, and beyond: Is COVID-19 less likely to spread in rich neighborhoods?

**DOI:** 10.1371/journal.pone.0267487

**Published:** 2022-05-10

**Authors:** Yue Gong, Guochang Zhao

**Affiliations:** 1 School of Urban Design, Wuhan University, Wuhan, Hubei, China; 2 Research Institute of Economics and Management, Southwestern University of Finance and Economics, Qingyang District, Chengdu City, China; Xiamen University, CHINA

## Abstract

Since December 2019, the COVID-19 pandemic has quickly spread across the world. The traditional understanding of the relationship between wealth and the spread of contagious diseases is that similar to many precedent epidemics, the pandemic spread easily in poor neighborhoods in many countries. The environmental and socioeconomic implications of the COVID-19 pandemic are still poorly understood, thus this paper examines the relationship between neighborhood characteristics and the spread of the pandemic through a case study of Shenzhen, a Chinese megacity with many low-income rural migrants. The major finding is that wealthier and larger neighborhoods in Shenzhen were more likely to be infected in the first wave of the pandemic in 2020. This spread pattern is likely to result from China’s strict control to prevent the pandemic, human mobility, and demographic characteristics such as income. This finding reveals a new phenomenon that contrasts with the traditional understanding of the influence of wealth on the spread of epidemics. This paper enriches the understanding of the role of neighborhoods in the spread of the pandemic, and it has important public policy implications.

## Introduction

On December 31, 2019, the first COVID-19 infection was reported in the city of Wuhan in Hubei Province, China [1, 2]. Even though the Chinese government locked down Wuhan on January 23, 2020, the epicenter of the pandemic, the pandemic had already spread across China [1–4]. In March and April 2020, Europe (especially Italy) and Iran were the most severely affected by the pandemic, and since April 2020, the virus has prevailed in the U.S. [5–7]. Since then, the virus has still impacted the entire world, raising great concern of academia. In addition to studying the attributes of the COVID-19 virus and vaccines, researchers have examined the spatiotemporal spread of the pandemic at global and national scales and demonstrated the uneven spatial spread associated with many socioeconomic and environmental factors [e.g., 1, 8–10].

As the pandemic continues to harm local communities, attention is turning from the global and national level to the local level such as the individual or neighborhood scale [11–14]. At a neighborhood scale, the COVID-19 spread has demonstrated distinctive socioeconomic patterns. In countries such as the U.S., Singapore, and many developing countries, poor neighborhoods where low-income people or ethnic minorities congregate have been more likely to be infected by COVID-19 [12, 14, 15]. For instance, in New York City, African Americans and their neighborhoods had higher numbers of infected cases and a higher risk of infection [11]. “Almost 93% of Singapore’s COVID-19 cases in the first 48 days occurred in dormitories for migrant workers,” and neighbors living in nearby areas were at greater risk of infection [15]. In the early stage of the pandemic in European countries such as Italy and Germany, infection rates were initially higher in wealthy districts but later the pandemic spread to poor neighborhoods, and richer districts then recorded fewer new infections [6, 13].

Neighborhoods and local communities can fight back when they are attacked by the virus [3, 13, 16]. For example, the governments of China and Italy sealed off neighborhoods to keep residents from being infected, and the Chinese government organized local institutions such as property management companies and homeowners’ associations and mobilized state employees and volunteers to enforce neighborhoods controls to prevent the spread [3]. In countries such as the U.S. and Brazil, some neighborhoods self-organized to mitigate the risk of infection through actions such as fundraising, food donation, cleaning efforts, accessing health services, and information dissemination [13, 16]. Neighborhoods have become the social foundation for community organization and the battlefield for public health authorities to arrest the spread of the pandemic.

The spread of COVID-19 infections still lack a comprehensive understanding [11, 15]. A few questions regarding the role of neighborhoods in the spread remain unanswered. For example, while poor neighborhoods are more vulnerable to infection, do wealthier neighborhoods, with better living conditions and more educated residents, experience reduced infection rates? What are the factors at a neighborhood level affecting the spread of the disease? Specifically, what is the spatial pattern of the spread across neighborhoods in China, and how is the pattern related to neighborhood characteristics such as socioeconomic status? These questions should be closely examined to understand and determine the importance of neighborhood characteristics in the pandemic.

This paper examines the spread of COVID-19 across neighborhoods, with a focus on the relationship between the spread and neighborhood socioeconomic characteristics, as indicated by average housing prices and property management fees. Based on the extant literature, this paper introduces a conceptual framework to analyze and understand the spread of COVID-19 while conducting on a case study of Shenzhen, a megacity in China. We find that larger, wealthier neighborhoods were more likely to be infected by COVID-19 in early 2020. On average, one log point increases in average housing prices and property management fees are respectively associated with a 127.1% increase and a 28.6% decrease in the odds ratio of infection. One log point increase in the number of buildings or apartments is associated with an increase in the odds ratio of infection ranging from 51.2–78.2%. In early 2020, richer people were more mobile and more easily expose to infection, bringing the virus into their neighborhoods. Chinese governments’ strict control largely prevented the COVID-19 spread including a further spread from wealthier neighborhoods to poor ones, resulting in the positive correlation between the spread and wealth. This paper also finds that neighborhoods with lower management fees and newer neighborhoods are more likely to be infected. The policy implications of this paper are that improved neighborhood governance efforts and the planning and development of smaller neighborhood units can help to slow the pandemic’s spread.

This paper expands the understanding of the pandemic and the practices that can be used to prevent the spread of COVID-19. First, the spread pattern in the relatively wealthy neighborhoods of Shenzhen is a new phenomenon unlike the spread of infection in poor neighborhoods in many other countries. Although because of the data limitation, we cannot strictly establish a causal relation from wealth to health, this finding enriches the experiences of the relationship between the distribution of wealth and the spread of contagious disease. Second, the conceptual framework introduced in this paper may guide the empirical analysis of the spread in future studies. Third, urban policies of improving neighborhood governance and planning and developing small-size neighborhoods can be grounded in this case study and thus contribute to promoting public health in neighborhoods.

The rest of this paper is organized into six sections. It begins with a theoretical overview of the major factors that influence the spread of COVID-19. In the third and fourth sections, the city of Shenzhen and the data are discussed, including a descriptive analysis. The methodology is introduced, and the empirical results of the analysis are provided in the fifth section. This is followed by the final two sections of discussion and conclusions.

## Impact factors of the COVID-19 spread

The spread of the pandemic is a complex process. The COVID-19 virus can infect many animals and is likely to be transmitted from animal hosts to humans [17, 18]. Nevertheless, the spread across the world is not simply affected by the attributes of the virus and species. The responses of diverse natural environments, different governments, societies, and public health systems can also greatly affect the spread [3, 9, 17]. The pandemic is a spatial process associated with both natural and human systems.

**Fig 1:**
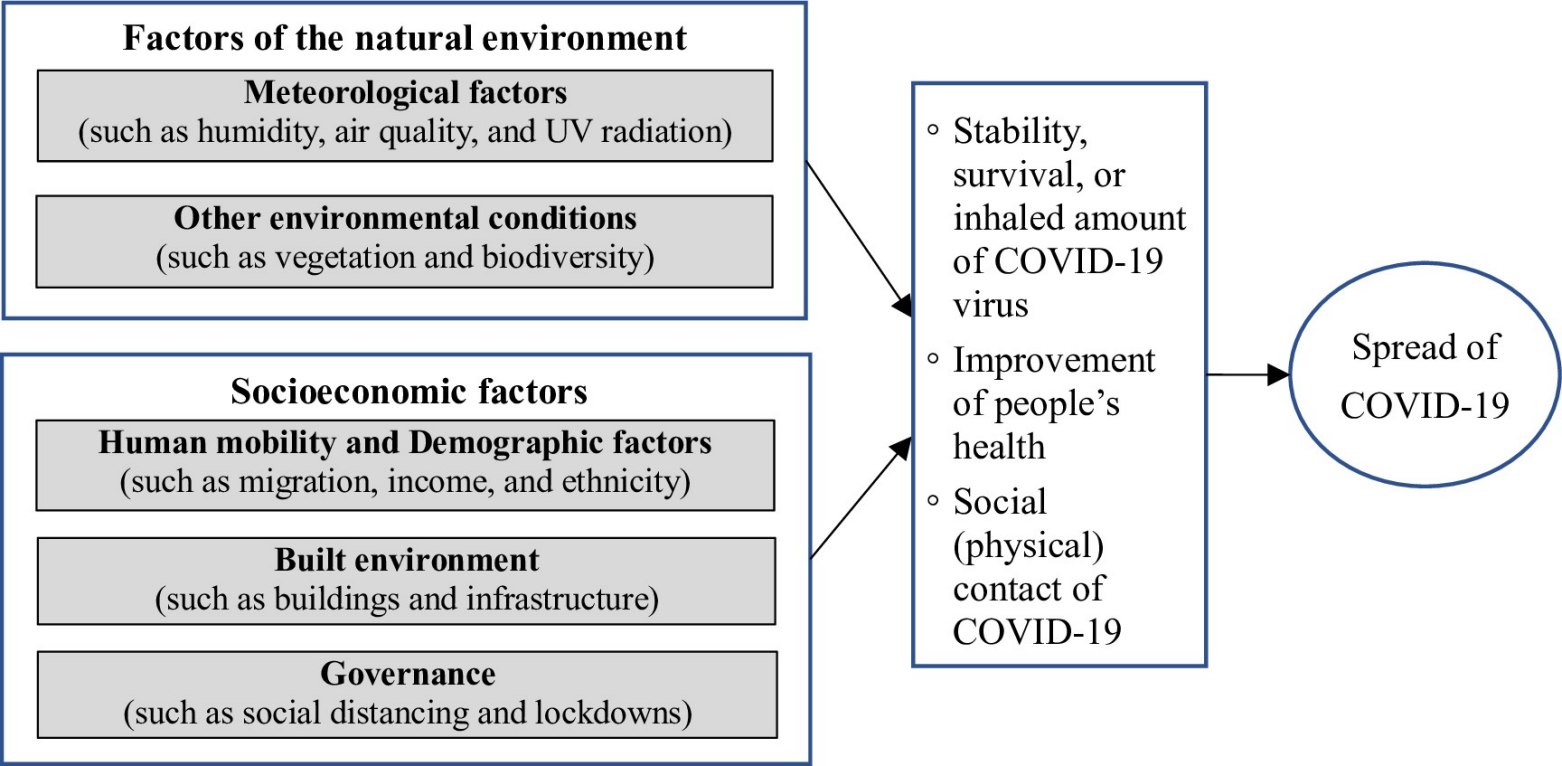
Important impact factors of the COVID-19 transmission.

The extant literature indicates that the spread has been impacted by the attributes of the virus and a diverse range of other factors, which can be categorized into two types: (1) factors of the natural environment and (2) socioeconomic factors related to human activity [3, 6, 7, 9, 11, 19, 20]. Fig 1 identifies the two types of key factors. Factors of the natural environment include meteorological factors (such as temperature, humidity, and air quality) and other natural, environmental conditions such as vegetation and biodiversity [2, 9, 19, 20]. Connolly, Roger [17] suggest three key socioeconomic factors that impact infectious diseases: demographic change, infrastructure, and governance. Likewise, the literature indicates that the socioeconomic factors affecting the COVID-19 transmission include demographic factors, built environment, and governance of socioeconomic systems [6, 14, 17, 21, 22].

In meteorological factors, low temperature, low humidity, and air pollution favor COVID-19 transmission [2, 19, 20]. Temperature and humidity can affect droplet stability in the atmosphere or the survival of viruses, impacting epidemic transmission [19, 20]. Through a study of COVID-19 cases in 3,235 regions across 173 countries, Carleton et al. find that ultraviolet (UV) radiation has a statistically significant effect on the COVID-19 spread [23]. Previous studies have shown that air pollution sources such as particulate matter (PM) can remain airborne for a long time, and infectious viruses attached to the PM can be inhaled and penetrate deep into lungs [2, 9]. Other environmental conditions such as vegetation and biodiversity are negatively associated with COVID-19 transmission [7, 24, 25]. Green space and biodiversity provide various ecological services that benefit physical and mental health and function as an infectious virus buffer [7, 24, 25].

Among the socioeconomic factors, human mobility and demographic factors such as ethnicity, income, and migration affect the spread of disease [9, 17, 26, 27]. Infectious diseases tend to afflict the poor and ethnic minorities disproportionately because they encounter more challenges to keep social distancing than richer people and have less access to resources to reduce the chance of becoming infected [6, 26]. In the current pandemic, they may lack access to medical services and basic living resources and typically do not have the option of working from home; they have no choice but to go to workplaces and face a greater risk of COVID exposure [14, 28]. Human mobility tied to demographic changes such as migration can significantly affect the spread [17, 22]. During early 2020, the COVID-19 virus in some European countries was imported by comparatively affluent travelers such as business people from China and ski tourists from the Alps [6, 13]. The travel quarantine of Wuhan stopped migration and significantly reduced (80%) case importations to other countries until mid-February [22].

The built environment (such as buildings and infrastructure) tied to people’s demography and mobility can influence infection [17, 21, 26]. For example, “COVID-19 risk is highest in more built-up, more walkable, and more physically deteriorated zip codes” in the U.S. [21]. In China, the density of point of interests around railway stations and travel time by public transport to activity centers were associated with the spread [29]. A review of 25 studies of the relationship between COVID-19 infection and built environment finds that “infection risk was positively associated with the density of commercial facilities, roads, and schools and with public transit accessibility” [30]. Yet, research has paid more attention to infrastructure and commercial areas than residences where, however, the infection often spreads.

Governance such as lockdowns and public health systems has shaped the spread of infectious diseases for centuries and has been used to control and prevent COVID-19 globally and locally [8, 9, 17]. As suggested by authorities such as the World Health Organization (WHO) [31], access to reliable information, washing hands frequently, and social distancing are crucial measures to help individuals remain healthy. Many governments around the world have applied social distancing and a range of governance policies such as travel bans, neighborhood closures, and restrictions on gathering [3, 8, 14]. Social distancing has widely proved to be effective in reducing people’s social contact and fighting the pandemic [3, 14]. A study of 1700 policy interventions in six countries including China, Iran, and the U.S. finds that although imposing large costs on society, these policies mentioned above have achieved large, beneficial, and measurable health outcomes, averting approximately 495 million total infections [32].

A neighborhood is a residential community having fairly uniform socioeconomic characteristics throughout [14, 16, 33]. In the neighborhood unit model, a neighborhood is an area of a quarter-to-half-mile radius with residential buildings, roads, retailers, and institutions such as a community center and an elementary school [33]. As discussed before, neighborhood characteristics such as neighborhood governance and residents’ income levels and socioeconomic status are tied to COVID transmission. Built environments of neighborhoods also impact the spread. Low-income people live in built environments that lack adequate shelter, adequate sleeping spaces, and basic infrastructures such as communication media, toilets, sewage, and running water; thus, they are easily attacked by the COVID-19 virus [16, 28]. A study of 164 million street-view images in the U.S. indicates that the built environment of neighborhoods such as building densities, the walkability of sidewalks, and physical conditions affect residential density, human mobility, and therefore social distancing, all of which affect residents’ chances of contacting the virus [16]. Neighborhood socioeconomic characteristics determine how neighborhoods operate in a crisis such as the pandemic and how their residents respond to and are impacted by the pandemic [14, 16].

In short, the spread of COVID-19 has been affected by two types of factors: factors of the natural environment including meteorological and other environmental factors, and socioeconomic factors including demographic factors, human mobility, the built environment, and governance. Environmental factors can affect the stability, survival, and inhalation of the COVID-19 virus and improve people’s health; socioeconomic activities mainly affect people’s social and physical contact and the risk of inhaling the virus, ultimately impacting the spread (Fig 1). A neighborhood is the basic spatial and socioeconomic unit influencing COVID-19 transmission. Thus, a better understanding of neighborhoods can help analyze, predict, and control the pandemic.

## Background: Shenzhen in the pandemic

This paper carries out a case study of Shenzhen, a Chinese megacity located in South China (see Fig 1). Shenzhen is quite suitable for this research for at least three reasons. First, Shenzhen has diverse neighborhood conditions in terms of demographic and socioeconomic characteristics. In 2018, Shenzhen’s gross domestic product (GDP) per capita was 189,568 *yuan*, which was almost three times that of China [34, 35]. There are many middle-class and rich people and certainly many wealthy neighborhoods in Shenzhen. Shenzhen is also a migrant city: In 2018, of the 13 million people in Shenzhen, 8.5 million were migrants [34]. The majority of Chinese migrants are the low-income rural people who come from the countryside to the city and usually live in urban villages (villages-in-the-city or *chengzhongcun*) or dormitories with low-quality living conditions [36]. The variations in neighborhood attributes make quantitative analysis practicable.

Second, the infection cases in Shenzhen provide sufficient information for analysis. The cases in Shenzhen rapidly increased until late January 2020 and then quickly decreased [18, 37]. By the end of April when the spread was controlled, there were 423 cases over 249 addresses in Shenzhen [38, 39]. The number of cases is not enormous relative to the city’s population but sufficient for this research. The complete addresses of cases were released to the public; thus, this paper can compare neighborhoods with and without cases and identify the environmental and socioeconomic attributes associated with COVID-19 infections.

Third, Shenzhen is typical of the strict policies used to prevent the spread of COVID-19. While locking down Hubei Province including Wuhan, the Chinese government enforced strict travel control across the country between late January and April, 2020 [22]. “By 29 January, all provinces across China had launched the highest level of response for major public health emergencies” [18]. The response includes strict monitoring, reporting, and quarantine, as well as rigid social distancing such as canceled or suspended public and economic activities in many provinces and cities including Shenzhen [18]. In Guangdong, any suspected cases were immediately isolated for further medical treatment [40]. To fight the pandemic, China exerted extreme control over public life [8].

Like many other Chinese cities, Shenzhen applied strict governance for several months. It implemented rigorous inspections in both transportation hubs and neighborhoods [41]. As soon as the pandemic broke out, the government regulated all neighborhoods uniformly, including the enclosure of all neighborhoods, the examination of body temperature of anyone who entered or left neighborhoods, and imposing 14-day isolation on anyone who came from pandemic areas such as Hubei [37, 42]. Similar measures against COVID-19 were applied to both urban villages where rural migrants congregated, and market housing compounds of the rich [41, 42]. After May 9, when the government of Guangdong downgraded these stringent controls [43], strict control gradually decreased.

The first author of this paper lived outside Shenzhen and commuted to Shenzhen for work in 2020. Before driving into the city and entering any neighborhood between February and April 2020, his body temperature was measured, approval was verified through online registration, and the permission of inspectors at checkpoints had to be granted. He observed that many urban villages were enclosed, and their main entrances were guarded. Before receiving a permit to enter his residential compound, he had to report his recent travel history and health conditions to a so-called grid (community) manager, who was actually a police officer. When the infection was spreading most quickly, the control of the city and its neighborhoods was rigid and similar to a lockdown.

## Data and variables

### 1. Data

To answer the research question in accordance with the conceptual framework presented in Fig 1, this study required information on the neighborhoods identified with each individual case of infection, and the two groups of factors (environmental and socioeconomic) which may affect the spread of COVID-19. Five data sources were used.

First, a list of the addresses where the confirmed cases of COVID-19 infection were located was extracted from UC Browser [38], a popular web browser available on smartphones. Since the outbreak of COVID-19, UC browser has collected information on COVID-19 from the government and other authorities and established a special channel of infoming the public about the pandemic. We downloaded a list of 249 addresses with infection cases in Shenzhen, of which nine are hotels, from the browser on April 29, 2020. Since then, the spread has been controlled and few new cases of infection were reported in Shenzhen in 2020. Because this paper focuses on residents rather than travelers and on neighborhoods rather than hotels, the nine observations in hotels were excluded from the sample.

The second data set contains neighborhood information retrieved from Lianjia (www.lianjia.com). The neighborhood variable identifies several different types of residential compounds, which include *xiaoqu* (market housing compounds), urban villages, *danwei* compound (work unit), and dormitory areas. *Xiaoqu* refers to gated market housing compounds. Urban villages are rural villages surrounded by rapidly urbanized areas. The *danwei* are the state work units, which were the dominant urban neighborhoods in Mao’s era. The boundaries of these neighborhoods are usually walls, gates, or roads; many of these neighborhoods are the approximate size of the neighborhood unit (Perry, 1929). There are only 36 *danwei* compounds and 33 dormitory areas in our final sample. Considering that the residents of these two types of neighborhoods tend to be similar and the limited number of observations, we classify *danwei* compounds and dormitories together as a single neighborhood type.

Lianjia is one of the largest real estate brokerage agencies in China. Its business includes housing transactions, leasing, decoration, and internet real estate finance. For every neighborhood listed on the website, Lianjia establishes a webpage that presents major neighborhood characteristics including average housing prices, attributes of the built environment, and the infrastructure and facilities surrounding the neighborhood. All of these are either the environmental or socioeconomic factors mentioned in Fig 1. Using a web crawler, we obtained data for 4,224 neighborhoods from the Lianjia website.

**Fig 2:**
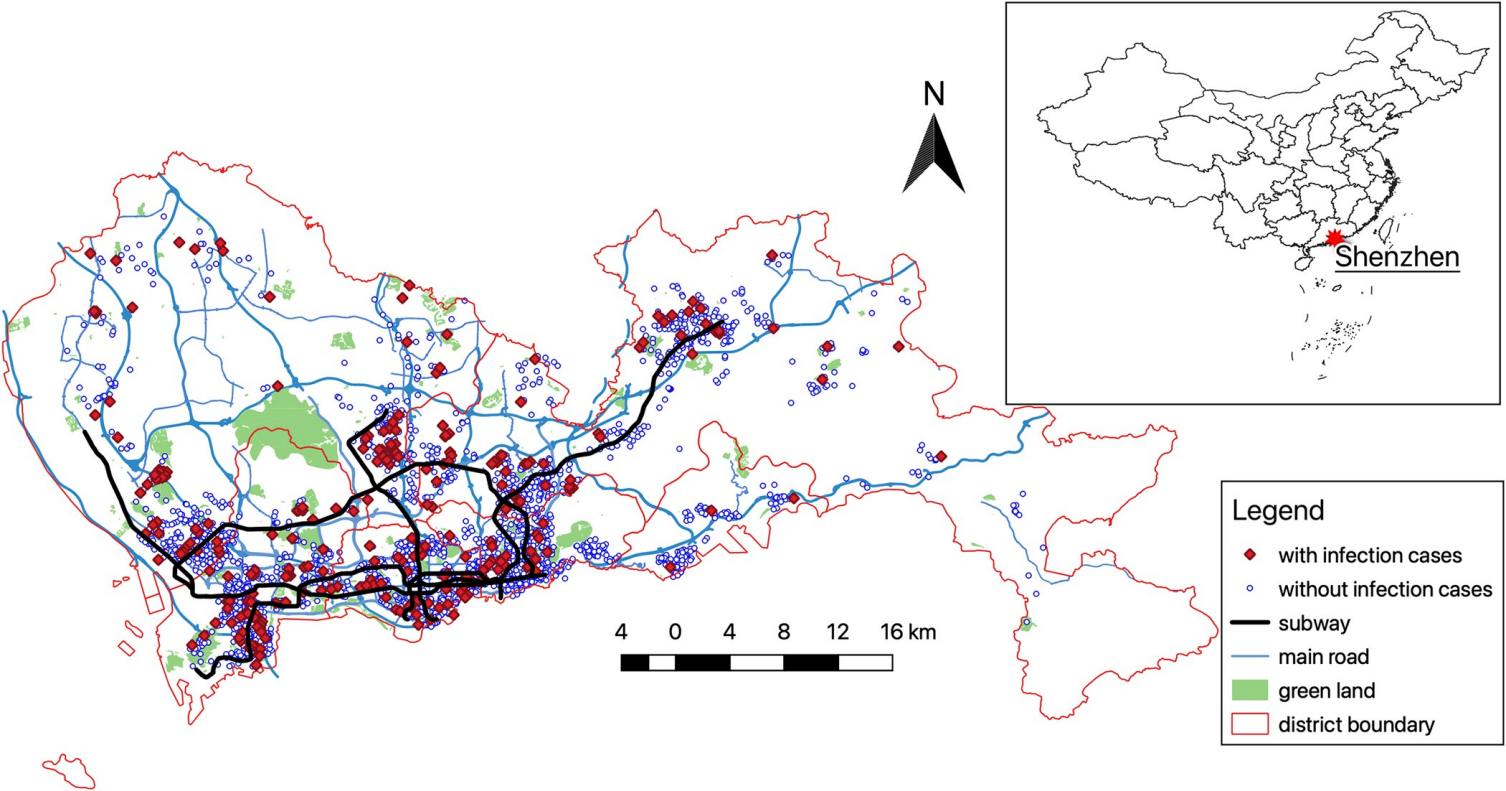
Neighborhoods with and without COVID-19 cases in Shenzhen.

By combining the two data sets, we found that approximately 6% of all neighborhoods in Shenzhen had COVID-19 cases. Then, we matched the two data sets by comparing their addresses and identified neighborhoods with confirmed cases. Fig 2 illustrates the geographic distribution of the neighborhoods: the infected and uninfected neighborhoods are concentrated in the south part of Shenzhen, which is the urban center.

We collected data from other sources. The third data set is daily temperature and humidity at the subdistrict scale, which were retrieved from Shenzhen Meteorological Data System (http://data.121.com.cn). Using QGIS 13.0, we matched the neighborhoods with Shenzhen’s subdistricts and estimated the corresponding temperature and humidity information of each neighborhood. The fourth data set is the monthly radiation from the European Center for Medium-Term Weather Forecasting ERA-interim dataset (https://www.ecmwf.int). Specifically, we chose the variable of surface net solar radiation of the ERA5 monthly averaged data. We also used QGIS 13.0 to calculate the average radiation within 500 meters of the neighborhood centroid. The fifth data set is the map of green space such as forested hills and meadows in Shenzhen, which was downloaded from the online map service provider BIGEMAP (http://www.bigemap.com).

### 2. Variables and sample selection

Average housing price, property management fee, and neighborhood size are the three variables of primary interest. To some extent, average housing price by itself captures the most important neighborhood characteristics including location, housing quality, nearby facilities, and the socioeconomic background of the residents [44, 45]. From the Lianjia website, we obtained two estimates of housing prices. The first type is the average list price in April 2020, and the second is the average transaction price during 2019. The 2020 list price is the ideal price sellers ask, and it is usually higher than the actual sale price. The 2019 transaction price is the actual sale price, and it is more accurate than the list price for measuring the socioeconomic status of neighborhoods and their residents. However, if there were no real estate transactions in a neighborhood during 2019, the value of the transaction price is not available. Because approximately one-third of all neighborhoods had no transactions during 2019, the method of using the transaction price in the data may lead to a problem with sample selection to some degree. To solve this problem, this paper uses the 2020 list price for the baseline analysis and the 2019 transaction price for a robustness check. In brief, neither of the two types of prices is perfect, but they are still good measures of neighborhood valuation.

Wealthy neighborhoods usually have high-quality management services [46]; thus they charge a higher property management fee. The property management fee is another indicator of the evaluation of neighborhoods and tends to vary with average housing prices. However, compared to housing price, which is in flux, the property management fee is usually stable over time. Once the property management fee is determined, it remains fixed for a long time (i.e., several years). In addition, compared to housing price, the property management fee reflects the quality of property management and is less dependent on factors such as location. During the pandemic, quality management is important while neighborhoods have implemented a series of policies to prevent infection. A neighborhood with a high management fee may do better in terms of sanitation, environmental sterilization, and community support. Because a uniform state of governance was applied across all neighborhoods in Shenzhen, we believe that there was no difference among the policies of these neighborhoods, and thus we did not select variables to measure it.

Under a widely applied quarantine, neighborhood size is another important factor associated with infection likelihood [47]. Given that Shenzhen is not the origin of the COVID-19 outbreak, larger neighborhoods are more likely to have cases of infection, as they usually have more residents. Here, we use the number of buildings and apartments (households) as two measures of neighborhood size.

In addition, we selected three groups of control variables. The first group refers to other neighborhood characteristics in dummy variables that indicate built years, urban village and *danwei* compound/dormitory. These neighborhood attributes are correlated with the residents’ demographic and socioeconomic characteristics. The second group includes factors of the built environment: availability of nearby public infrastructure and commercial facilities including subway stations, bus stops, primary schools, kindergartens, supermarkets and pharmacies within 1500 meters, and hospitals and parks within 2000 meters. The 1500- and 2000-meter thresholds were used by the Lianjia website. The third group includes environmental factors: average temperature, humidity and radiation from January to June, and the area of green space within 500 meters of the neighborhood centroid.

After excluding neighborhoods with missing values for both the 2020 list price and the 2019 transaction price, built year, or property management fee, the final data set had 3010 observations.

## 3. Results

### 3.1 Summary statistics and descriptive analyses

**Fig 3:**
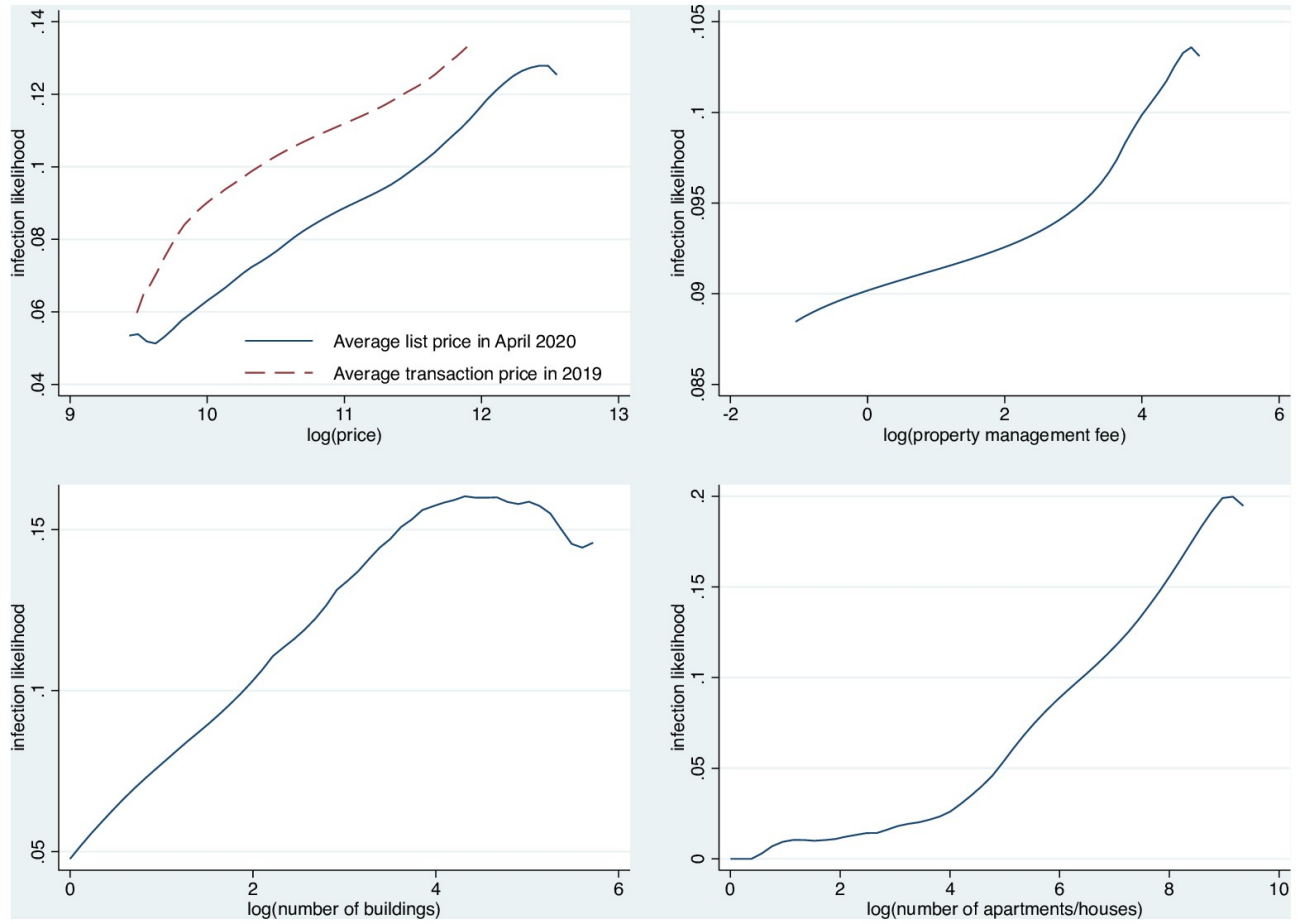
Relationship between infection likelihood and housing price, property management fee, and neighborhood size. Note: The relationships are estimated through kernel-weighted local smoothing.

Table 1 presents summary statistics for neighborhoods with and without cases of COVID-19. Panel A lists neighborhood characteristics. First, 2737 of the 3010 neighborhoods have COVID-19 cases and 273 neibhborhoods do not have COVID-19 cases–nine percent approximately. Second, it shows that the neighborhoods with cases have higher average housing prices than those without cases. The differences between the list and transaction prices are approximately 25% of the standard deviation. Thus, relatively rich people, living in wealthy neighborhoods, appear more likely to be infected in Shenzhen. Third, on average, neighborhoods with cases have 5.36 more buildings and 466 more apartments than those without cases. This result is consistent with our expectation that larger neighborhoods are more likely to have cases. Fig 3 shows the non-parametrically estimated unconditional relationships between infection likelihood and each of the three measures (housing price, property management fee, and neighborhood size). This figure suggests that infection likelihood increases linearly with the log of the average housing price, property management fee, and neighborhood size, which provides insight for the specification of an empirical model. In addition, Table 2 presents the correlation coefficients between housing price, property management fee, and neighborhood size. It shows that only the two types of housing prices are highly correlated, while other variables do not have high pair-wise correlation. Thus, these three measures contain adequately independent neighborhood characteristics.

**Table 1:** Summary statistics: Neighborhoods with and without COVID-19 cases.

	Without infection cases			With infection cases			Diff.
Variables	Mean	S.D.	N	Mean	S.D.	N	(4)-(1)
	(1)	(2)	(3)	(4)	(5)	(6)	(7)
**Panel A: Neighborhood characteristics**							
Average list price in April 2020 (yuan/m^2^)	66974	31264	2724	75103	31621	273	8128**
Average transaction price in 2019 (yuan/m^2^)	58964	22870	1746	64472	23991	216	5508**
Property management fee (yuan/m^2^ per month)	2.92	3.16	2737	3.03	1.57	273	0.11
Number of buildings	7.63	14.06	2737	12.99	23.79	273	5.36**
Number of apartments/houses	627	681	2737	1093	831	273	466**
Built year: 1980s and before (1, yes; 0, no)	0.09	0.29	2737	0.06	0.24	273	-0.03*
Built year: 1990s (1, yes; 0, no)	0.34	0.47	2737	0.20	0.40	273	-0.14**
Built year: 2000s (1, yes; 0, no)	0.41	0.49	2737	0.45	0.50	273	0.05
Built year: 2010s (1, yes; 0, no)	0.16	0.36	2737	0.29	0.45	273	0.13**
Urban village (1, yes; 0, no)	0.04	0.19	2737	0.04	0.20	273	0.00
*Danwei* compound/dormitory (1, yes; 0, no)	0.02	0.15	2737	0.01	0.09	273	-0.02*
**Panel B: Factors of natural environment**							
Average temperature from January to June (°C)	22.97	0.51	2726	23.08	0.42	273	0.10**
Average radiation from January to June (million joules per square meter per day)	11.66	0.31	2724	11.55	0.35	272	-0.12**
Average humidity from January to June (% rh)	77.38	1.96	2726	77.22	1.82	273	-0.16
Area of green space within 500m (m^2^)	0.07	0.14	2737	0.07	0.14	273	0.00
**Panel C: Nearby public infrastructure and commercial facilities**							
Number subway stations	4.45	3.17	2737	3.73	3.06	273	-0.73**
Number of bus stops	7.42	2.77	2737	7.57	2.47	273	0.14
Number of kindergartens	4.77	1.00	2737	4.81	0.93	273	0.04
Number of primary schools	4.43	1.32	2737	4.18	1.53	272	-0.26**
Number of hospitals	6.33	3.15	2737	5.76	3.06	272	-0.57**
Number of parks	7.96	2.13	2737	7.84	2.04	273	-0.12
Number of supermarkets	6.48	1.56	2737	6.58	1.36	273	0.10
Number of pharmacies	4.75	1.03	2737	4.78	0.94	273	0.03

Note: 2737 of the 3010 neighborhoods have COVID-19 cases and 273 do not have COVID-19 cases–nine percent approximately. Significance codes

*** p<0.01

** p<0.05

* p<0.1.

**Table 2:** Correlation between housing price, property management fee, and neighborhood size.

	Average list price in April 2020	Average transaction price in 2019	Property management fee	Number of buildings	Number of apartments/houses
Average list price in April 2020	1.00				
Average transaction price in 2019	0.95	1.00			
Property management fee	0.15	0.13	1.00		
Number of buildings	0.01	-0.02	-0.04	1.00	
Number of apartments/houses	0.03	0.04	0.07	0.31	1.00

Table 1 also shows that residents in older neighborhoods and *danwei* are less likely to be infected, probably because there are usually more local residents in these areas thus less human inflow from Hubei Province or other epidemic areas. Panel B reports the summary statistics of the natural environmental factors. Neighborhoods with infection cases are a little warmer but have less solar radiation than those without infection cases. Panel C demonstrates summary statistics of public infrastructure, and commercial facilities nearby neighborhoods. These estimates show that the numbers of subway stations, primary schools, and hospitals are negatively associated with the likelihood of infection, but other factors do not seem to be related to infection.

### 3.2 Empirical model and regression results

#### a) Model

Given that the dependent variable is binary (1 for the presence of cases and 0 for a lack of cases), we select a logit model:

$$P(y_{i}=1|x_{i})=\frac{exp\;(x_{i}^{\prime }\beta )}{1+exp\;(x_{i}^{\prime }\beta )}$$ (1)

where *y*_*i*_ is a binary variable indicating whether neighborhood *i* has COVID-19 cases, **x**_*i*_ is a vector of explanatory variables including neighborhood characteristics, environmental factors, public infrastructure and commercial facilities described in the previous section (the log is taken for housing price, neighborhood size, and property management fee).

The coefficients of the logit model are not very meaningful; thus, the odds ratio is usually used to interpret the results. Specifically, for the variable **x**_*ij*_, the odds ratio is defined as:

$$OR=\frac{P(y_{i}=1|x_{i})}{1-P(y_{i}=1|x_{i})}=\exp\;\left(x_{i}^{\prime }\beta \right),$$ (2)

which means the odds of infection are $\exp\;\left(x_{i}^{\prime }\beta \right)$ times the odds of non-infection in our case. Furthermore, the coefficient, for example, the *j*th variable’s coefficient *β*_*j*_, means that when **x**_*ij*_ increases by 1 unit, the initial odds ratio increases by the initial odds ratio multiplied by exp(*β*_*j*_)≈1+*β*_*j*_, or the odds ratio increases by approximately (100⋅*β*_*j*_) percent.

Here, we also conduct the probit model and linear probability model (LPM), which provided very similar results to those of the Logit model.

#### b) Baseline regression results

The results of baseline estimation are presented in Table 3. From columns (1) to (4), the controls are added step-by-step. As indicated in column (1), only housing price, property management fee, and neighborhood size are included. When these variables increase by 1 log point, the odds ratio of infection increased by 75%, 9.6%, 44.1%, and 93.4%, though the effect of property management fee is not statistically significant. This means that richer and larger neighborhoods are more likely to be infected by COVID-19. Overall, the results of estimation pertaining to housing price and neighborhood size are also consistent with the summary statistics in Table 1 and the unconditional relationship in Fig 3.

**Table 3:** The logit model results for COVID-19 infection odds ratio (OR).

Variables	(1)	(2)	(3)	(4)
Ln (Average list price in April 2020)	1.750**	2.304***	2.097***	2.623***
	(0.467)	(0.675)	(0.603)	(0.644)
Ln (Property management fee)	1.090	0.987	0.750**	0.711**
	(0.081)	(0.089)	(0.090)	(0.101)
Ln (Number of buildings)	1.441***	1.400***	1.553***	1.486***
	(0.137)	(0.138)	(0.136)	(0.162)
Ln (Number of apartments)	1.934***	1.847***	1.723***	1.767***
	(0.149)	(0.143)	(0.118)	(0.128)
Average temperature from January to June		0.000*	0.000**	0.000
		(0.000)	(0.000)	(0.000)
The squared average temperature from January to June		1.320*	1.390**	1.358*
		(0.211)	(0.222)	(0.247)
Average radiation from January to June		0.303***	0.431***	0.489*
		(0.107)	(0.135)	(0.182)
Average humidity from January to June		1.051	1.042	1.033
		(0.069)	(0.074)	(0.061)
Area of green space (m^2^)		1.091	1.126	0.798
		(0.525)	(0.515)	(0.250)
Built year: 1990s (1, yes; 0, no)			1.477	1.498*
			(0.383)	(0.348)
Built year: 2000s (1, yes; 0, no)			2.230***	2.128***
			(0.505)	(0.366)
Built year: 2010s (1, yes; 0, no)			3.291***	2.912***
			(0.641)	(0.558)
Urban village (1, yes; 0, no)			0.893	0.908
			(0.352)	(0.387)
*Danwei* compound/dormitory (1, yes; 0, no)			1.167	1.259
			(0.391)	(0.383)
Number of subway stations				0.970
				(0.030)
Number of bus stops				0.996
				(0.026)
Number of kindergartens				1.070
				(0.214)
Number of primary schools				0.788
				(0.147)
Number of hospitals				1.034
				(0.030)
Number of parks				0.911*
				(0.046)
Number of supermarkets				1.165
				(0.127)
Number of pharmacies				0.974
				(0.053)
Observations	2,996	2,996	2,996	2,996
F-stat for infrastructure & facilities				1883
p-value				0.000

Note: Robust standard errors, which are in parentheses, are clustered at the district level. Significance codes

*** p<0.01

** p<0.05

* p<0.1.

The environmental factors are added in column (2), which increases the magnitude and significance level of the effect of housing price, and has little influences on the effects of the property management fee and neighborhood size. Among the three environmental factors, the temperature has a nonlinear effect on the transmission and our case is in the part of positive correlation, although this relationship is only marginally significant, and the radiation has more significant effect statistically. In terms of the effects on odds ratio, they are larger and smaller than 1, respectively, for temperature and radiation. This is consistent with the finding in the descriptive analyses and may reflect a correlation between the transmission and population density. Fig 2 suggests that the neighborhoods in the central urban areas are more likely to spread COVID-19. The central urban areas with higher population densities are more likely to be slightly warmer because of the heat island effect, and usually receive less solar radiation because of more particles in the atmosphere, which can reflect some solar radiation back to the space and even absorb it.

Column (3) added the other neighborhood characteristics. The results for all variables remain similar compared to column (2) with the exception of property management fee. Surprisingly, the effect of property management fees becomes negative and significant. If the property management fee increases by 1 log point, the odds ratio of infection decreases by 25%. In addition, it also shows that the newer neighborhoods are more likely to have infections. Finally, neighborhood types have nothing to do with the likelihood. Specifically, compared with other types of neighborhoods, urban village and *danwei* compound/dormitory do not have significantly different likelihood with infection cases.

In column (4), we add the variables of public infrastructure and commercial facilities. The inclusion of these variables in the model only slightly changed the coefficients of neighborhood characteristics. The main difference is that the magnitudes of average housing prices increase even larger– 1 log point increase in housing price is now associated with approximately 162% more likely to have COVID-19 infection cases. The public infrastructure and commercial facilities are not statistically significant, which is not consistent with the results of previous studies [e.g., 17, 21]. One explanation is that infrastructure and commercial facilities are homogenously distributed thus there is not enough variation to lead to significant estimators. An alternative explanation is that these variables are highly correlated, which causes multilinearity and insignificance. To test the two explanations, a joint significance test was conducted (the F-statistics at the bottom of Table 2). The results show that at least one variable of the infrastructures and facilities is significantly associated with the odds of reported infection. Nevertheless, whatever is the reason for the insignificance, this variable does not affect the association between the likelihood of infection and the explanatory variables of our primary interests–housing price, property management fee, and size of neighborhood.

#### c) Robustness and heterogeneity tests

The results of a robustness test of the logit model are shown in Table 4. The 2020 average list price was substituted for the 2019 average transaction price, which causes the sample size to decrease by approximately 1000. Columns (1)–(4) in Table 4 have the same model specifications as those in Table 3, but the magnitude of the coefficient of average housing prices increases substantially. Taking column (4), the fully specified model, as an example, one log point increase in average housing prices is associated with a 171% increase in the odds ratio of infection, while the corresponding effect in Table 2 is 127.1%.

**Table 4:** Robustness tests.

Variables	(1)	(2)	(3)	(4)	(5)
Ln (Average transaction price in 2019)	1.803*	2.568***	2.401**	3.187***	
	(0.582)	(0.937)	(0.819)	(0.880)	
Ln (Average list price in April 2020)					2.888***
					(0.655)
Property management fee	Yes	Yes	Yes	Yes	Yes
Neighborhood size	Yes	Yes	Yes	Yes	Yes
Factors of natural environment	No	Yes	Yes	Yes	Yes
Other neighborhood characteristics	No	No	Yes	Yes	Yes
Public infrastructure and commerce facility	No	No	No	Yes	Yes
Observations	1,949	1,949	1,932	1,932	1,932

Note: Robust standard errors, which are in parentheses, are clustered at the district level. Significance codes

*** p<0.01

** p<0.05

* p<0.1.

Since Tables 3 and 4 have different samples, one may wonder what causes these differences. Do these differences result from the different measures of housing price or from differences in sample composition based on the two types of housing price? To answer this question, another estimation was conducted and demonstrated in column (5) in Table 4: we used the 2020 list price, but we selected only the samples with non-missing values of the 2019 transaction price. The result in column (5) of Table 3 is between that in column (4) of Table 3 and that in column (4) of Table 4. Thus, the difference between Tables 3 and 4 is partially due to the different samples, and our results are robust to the choice of housing price estimates.

**Fig 4:**
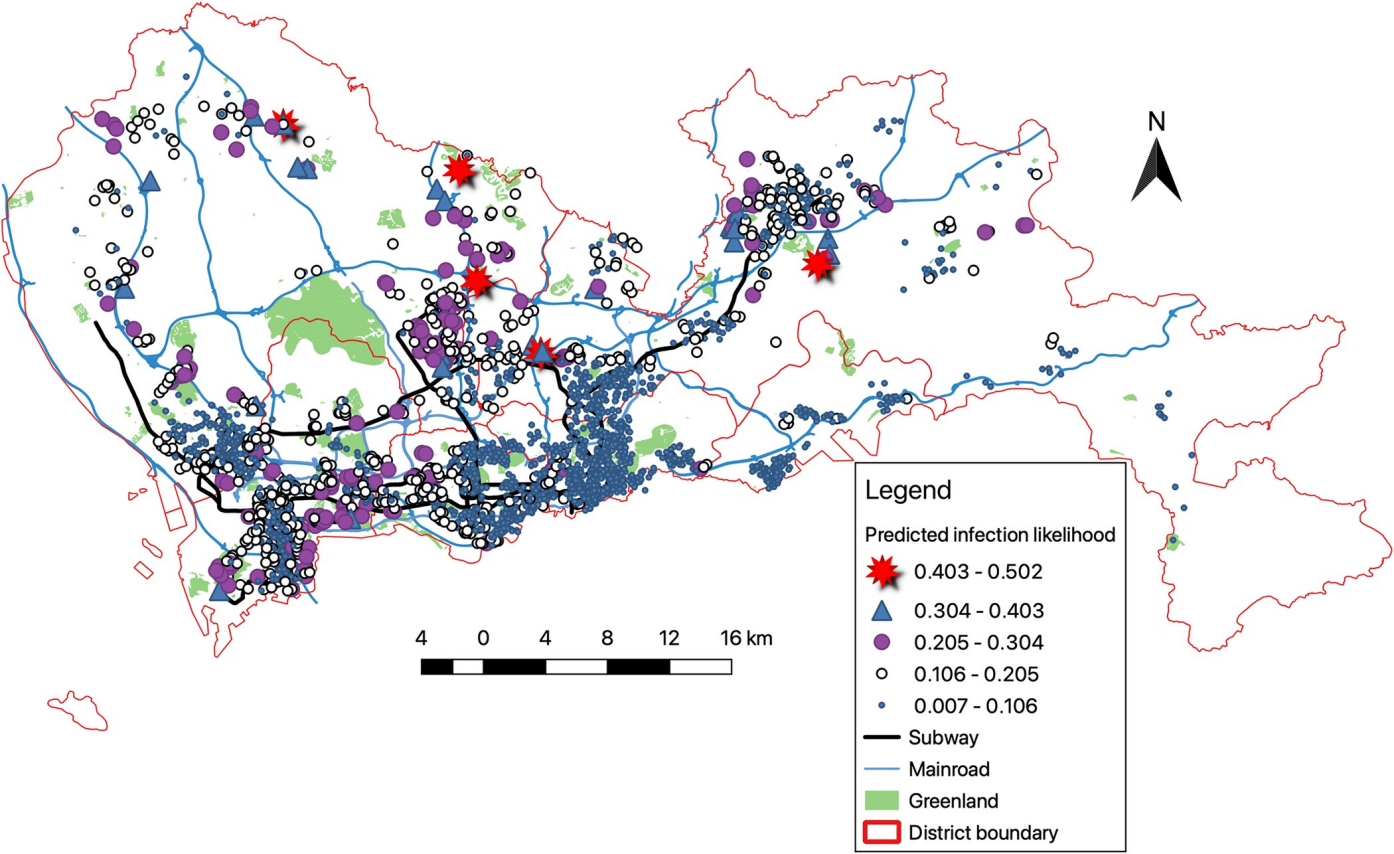
The predicted likelihood of COVID -19 infection using the average 2020 list price.

Because the COVID-19 outbreak happened before late January 2020, many migrants who worked in Shenzhen had returned to their hometowns before the breakout, because of the Chinese Spring Festival. Thus, accurate neighborhood demography data were not available. Therefore, this paper cannot examine detailed differences between neighborhoods with and without the 2019 transaction price. However, regarding the prediction of infection likelihood, the differences have no substantial influence on the likelihood. S1 Fig in S1 Appendix shows the infection likelihood predicted by the logit model with two different types of prices that are highly correlated. Fig 4 demonstrates the geographic distribution of the predicted infection likelihood using the logit model reported in column (4) of Table 3. The neighborhoods predicted to have infections are concentrated in the south part of Shenzhen, which is similar to the reported infection pattern portrayed in Fig 2.

Table 5 shows the results of the heterogeneity tests. The interaction terms between any two variables of the three primary factors—housing price, property management fee, and neighborhood size—are included in Panel A to investigate whether these variables affect the marginal effect of other variables on infection likelihood. All interaction terms are insignificant. The results indicate that these three variables do not significantly amplify the effect of each other on infection likelihood.

**Table 5:** Heterogeneity tests.

Variables	(1)	(2)	(3)	(4)	(5)
**Panel A: Heterogeneity over housing price, property management fee, and neighborhood size**					
Ln (Average list price in April 2020)	2.411***	0.503	2.6051***	2.6161***	2.663**
	(0.703)	(0.614)	(0.657)	(0.630)	(1.079)
Ln (Property management fee)	0.708**	0.725**	0.531**	0.861	0.825
	(0.098)	(0.105)	(0.154)	(0.823)	(1.950)
Ln (Number of buildings)	0.896	1.5081***	1.293**	1.4811***	1.4861***
	(1.081)	(0.162)	(0.136)	(0.178)	(0.164)
Ln (Number of apartments)	1.7741***	0.106	1.8141***	1.8201***	1.7661***
	(0.129)	(0.199)	(0.130)	(0.337)	(0.127)
Ln (List price)*Ln (No. of buildings)	1.046				
	(0.118)				
Ln (List price)*Ln (No. of apartments)		1.288			
		(0.221)			
Ln (PMF)*Ln (No. of buildings)			1.143		
			(0.155)		
Ln (PMF)*Ln (No. of apartments)				0.970	
				(0.148)	
Ln (list price)*Ln (PMF)					0.987
					(0.209)
**Panel B: Heterogeneity across built years**					
Ln (Average list price in April 2020)	3.488	2.6821***	2.6641***	2.7371***	
	(4.315)	(0.725)	(0.652)	(0.666)	
Ln (Property management fee)	0.716**	0.584	0.6921***	0.6741***	
	(0.107)	(0.271)	(0.094)	(0.097)	
Ln (Number of buildings)	1.4821***	1.4991***	1.047	1.4991***	
	(0.162)	(0.158)	(0.148)	(0.165)	
Ln (Number of apartments)	1.7661***	1.7551***	1.7981***	1.013	
	(0.121)	(0.129)	(0.121)	(0.166)	
Built year: 1990s	100.977	1.364	0.680	0.0631***	
	(1,594.745)	(0.320)	(0.459)	(0.068)	
Built year: 2000s	148.642	1.8261***	0.651	0.0121***	
	(1,879.183)	(0.382)	(0.432)	(0.020)	
Built year: 2010s	18.921	6.1421***	1.252	0.147	
	(265.124)	(3.770)	(0.709)	(0.198)	
Ln (list price)* Built year: 1990s	0.686				
	(0.964)				
Ln (list price)* Built year: 2000s	0.685				
	(0.766)				
Ln (list price)* Built year: 2010s	0.847				
	(1.054)				
Ln (PMF)* Built year: 1990s		1.362			
		(0.731)			
Ln PMF)* Built year: 2000s		1.380			
		(0.737)			
Ln (PMF)* Built year: 2010s		0.687			
		(0.413)			
Ln (No. of buildings)* Built year: 1990s			1.312		
			(0.260)		
Ln (No. of buildings)* Built year: 2000s			1.602**		
			(0.352)		
Ln (No. of buildings)* Built year: 2010s			1.353		
			(0.288)		
Ln (No. of apartments)* Built year: 1990s				1.6421***	
				(0.239)	
Ln (No. of apartments)* Built year: 2000s				2.2391***	
				(0.574)	
Ln (No. of apartments)* Built year: 2010s				1.621**	
				(0.333)	
Observations	2,996	2,996	2,996	2,996	2,996

Note: All regressions control for the environmental factors, public infrastructure and commercial facilities. Robust standard errors, which are in parentheses, are clustered at the district level. PMF refers to property management fee. Significance codes

*** p<0.01

** p<0.05

* p<0.1.

Panel B shows the results of the interactions between built years and each of the three variables of primary interest. The interactions in columns (1) and (2) are not significant, but some of those in columns (3) and (4) are significant. They indicate that larger neighborhoods built in the 2000s are more likely to have infection cases compared to those built in other periods. In addition, larger neighborhoods built in the 1990s and 2010s have no significant difference.

## Discussion: Explaining the spread and policy implications

The examination of the case of Shenzhen demonstrates that wealthier neighborhoods were more likely to be infected. This significant positive relationship between the COVID-19 spread and the socioeconomic characteristics of neighborhoods contrasts with the global phenomena of the spread and the traditional experience of epidemics. This spread pattern in Shenzhen differs from not only many countries (e.g., the U.S., Singapore, and many developing countries) where the pandemic spread across poor neighborhoods and but also some European countries where the spread appeared in richer areas but quickly across poor neighborhoods [6, 13]. The spread in wealthier neighborhoods also contrasts with the traditional understanding that infection usually spread quickly in poor neighborhoods.

This unique spread pattern in Shenzhen should be shaped by special factors according to the framework of COVID-19 transmission in Fig 1. Strict state governance applied to prevent the spread can significantly impact the COVID-19 infection at the neighborhood level. While the entire province of Hubei was locked down, ordinary people could not leave or enter in early 2020 [8, 48]. Strict controls were applied to isolate neighborhoods and constrain human mobility within provinces and cities, increasing social distance and reducing infection risks. The strict control of human mobility and neighborhoods greatly reduced the mobility of low-income rural migrants. The travel ban, monitoring, and control of population inflows started in late January of 2020. According to Shenzhen News [41], five million people had left Shenzhen by late January, 2020. Due to the travel ban and migration control, rural migrants could not travel by train or bus for long-distance travel and remained in their hometowns after the Chinese New Year period. After February, migrants gradually returned to Shenzhen. When entering Shenzhen, they were subjected to inspection and a possible 14-day quarantine.

By comparison, more affluent people had the capital to increase their mobility, in particular in the beginning of the COVID-19 outbreak. For example, of the 25 infected people detected in Shenzhen on February 5 and February 6, 2020, 16 drove their cars from other cities including Wuhan to Shenzhen, and 6 people returned to Shenzhen from overseas [49]. Most rural migrants do not have private cars and cannot travel overseas, but the wealthy can more easily travel across the country and thus bring COVID-19 to Shenzhen. With more physical mobility and interaction than rural migrants, the affluent were more likely to bring the virus into their neighborhoods. However, the infection was not spread over a neighborhood, indicated by the fact of only 423 cases over 249 addresses in Shenzhen [38, 39]. Due to the strict control, the affluent and other infected people were quarantined as soon as their infection were detected. Likewise, infection hardly spread across neighborhoods or from wealthy neighborhoods to migrants’ poor neighborhoods and the spread was finally prevented in April, 2020.

Another explanation may be the “healthy immigrant effect”: Immigrants have better health status than the natives and are less likely to be infected [50, 51]. This explanation is hardly to be examined because we do not have micro data to isolate these two different hypotheses: mobile ability vs. initial health status. Nevertheless, Shenzhen is a new city: Forty years ago, it was only a small town with a native population of no more than 0.3 million and grew up to a megacity with 175.6 million residents in 2020. From this perspective of urbanization, almost all residents including those infected and uninfected in Shenzhen in early 2020 are migrants. Thus, the mobility explanation is much more reasonable.

To sum up, the empirical results do not imply that higher housing prices and larger neighborhoods *cause* a higher likelihood of COVID-19 infection, and the positive association should not be considered a causal effect. Although quantitative data on neighborhood governance are not available, in addition to the analysis above, studies have proved that strict control of human mobility and social distancing are very effective to prevent the spread [4, 10, 22, 27]. Thus, the spread of the virus may result from the strict control of state governance, residents’ mobility, and their demographic characteristics such as higher income. Unlike wealthier neighborhoods in many other countries where better medical services and healthier living environment reduce infection risks, wealthier neighborhoods in China housed richer residents who, with higher mobility, had more chances of exposing to COVID-19 and were more easily infected in early 2020.

The findings provide three points of insight into the limited understanding of the COVID-19 spread at micro spatial scale and policy implications to prevent the spread. First, strict neighborhood governance can be very effective in fighting the pandemic. These governance strategies include 14-day quarantine of any suspected person of infection, enclosure of all neighborhoods, an examination of body temperature of anyone who entered or left neighborhoods, and mobilization of state employees, property management companies, and community volunteers in implementation. The empirical results indicate that, during the period of greatest COVID-19 spread (January to April 2020), a potential quick COVID-19 spread in poor neighborhoods were eliminated in Shenzhen. Other countries may not apply strict governance, and Omicron has decreasing negative impacts on health than preceding COVID variants. Nevertheless, the states, neighborhoods, and grass-roots organizations in other countries can learn from Shenzhen to apply governance methods such as the examination of body temperature and mobilization of communities to maintain social distancing and hygienic conditions.

Second, quality property management can help prevent the infection. Given the negative correlation between property management fees and infection likelihood, infection risks may be reduced for someone who can move into a neighborhood charging a higher property management fee. An increase in the fee may also reduce the risk because the increase may improve the management quality in the neighborhoods in terms of public health. Governments may subsidize neighborhood property management companies to maintain good conditions of public health.

Third, the planning and development of neighborhoods with smaller blocks can reduce the size of neighborhoods and therefore reduce the risk of COVID-19 infection. Neighborhoods with more buildings and apartments, where people are more easily infected, should receive more attention and resources to prevent infection. More importantly, the pandemic can easily spread in China’s superblock neighborhoods; thus, appropriate measures of urban planning and design should be taken. Superblock neighborhoods are common in Chinese cities. The superblocks result from a few serious problems such as land speculation and resident relocation and have caused a policy debate in planning research and practices [52]. The Chinese government and some scholars have advocated the development of smaller neighborhoods in the future [52, 53]. This study provides support to developing smaller neighborhoods from the perspective of public health.

## Conclusions

The relationship between the COVID-19 pandemic and neighborhood characteristics has received scholarly attention [e.g., 3, 12, 16]. However, the understanding of this relationship is still limited. This paper examines what kind of neighborhoods are more likely to be infected by COVID-19. Based on the extant literature, it summarizes a theoretical framework of the factors that influence the spread of COVID-19 to guide analysis (Fig 1). This framework is tested in a case study of Shenzhen, which is typical of the strict public health controls widely implemented in Chinese cities to fight the pandemic, for analysis.

This paper finds that wealthier and larger neighborhoods in Shenzhen were more likely to be infected in the pandemic in 2020. Specifically, one log point increase in average housing prices and property management fees are associated with a 127.1% increase and a 28.6% decrease in the odds ratio of infection, respectively. One log point increase in the number of buildings or apartments is associated with an increase in the odds ratio of infection ranging from 51.2–78.2%. This finding contrasts with the traditional understanding that poor neighborhoods are more easily infected. The major reason is likely to be China’s strict control to prevent the pandemic and wealthier residents’ higher mobility. Richer people were more mobile and more easily exposed to infection, bringing the virus into their neighborhoods; nevertheless, Chinese governments’ strict control largely prevented the COVID-19 spread including a further spread from wealthier neighborhoods to poor ones.

This paper also finds that neighborhoods with lower management fees and that were built more recently are more likely to be infected. An increase of management fees may improve the quality of neighborhood management in terms of public health. Commercial facilities including supermarkets, restaurants, and pharmacies have few significant impacts on the infection. Public infrastructure such as subway stations, kindergartens, primary schools, and hospitals are likely to have few impacts. However, this result may be due to multicollinearity and requires further analysis when more data are available.

Unlike previous findings that environmental factors such as weather, humidity, and green space impact the spread, most of these factors are not significant at the neighborhood scale. An explanation is that these factors change so little at the neighborhood scale that the variance of their impacts on different neighborhoods may be negligible, but further analysis is still needed.

This paper contributes to the understanding of the COVID-19 spread and policy implications in three points. First, this paper reveals a new spatial spread pattern, which contrasts with our traditional understanding of the relationship between inequity and epidemics. It suggests that the spread pattern possibly results from human mobility, income, and strict control of migration and neighborhoods, which helps to further the studies of governing the pandemic when more governance data at the neighborhood level are available. Second, the theoretical framework of the spread can be applied and further enriched in future research. Third, the findings of this paper suggest that strict neighborhood governance and the planning and development of small neighborhoods are effective strategies to fight the COVID-19 pandemic.

## Supporting information

S1 Appendix (DOCX)

S1 Data (DTA)
