# Supplementary material for: Wealth, health, and beyond: Is COVID-19 less likely to spread in rich neighborhoods?
Source: PLoS One. 2022 May 10;17(5):e0267487. doi: 10.1371/journal.pone.0267487 (PMC9089870; doi:10.1371/journal.pone.0267487)
Supplement: S1 Appendix — (DOCX) [file pone.0267487.s001.docx]

**S1 Fig The predicted infection likelihood using list and transaction prices**
